# Long-Term Bowel and Urinary Function Outcomes and Quality of Life in Patients with Anorectal Malformations: 20 Years of Experience

**DOI:** 10.3390/children12081042

**Published:** 2025-08-08

**Authors:** Fabio Baldanza, Francesco Grasso, Marco Pensabene, Maria Sergio, Maria Rita Di Pace

**Affiliations:** Pediatric Surgical Unit, Department Health Promotion of Mother and Child Care Internal Medicine and Medical Specialties, University of Palermo, 90127 Palermo, Italy; francesco.grasso@policlinico.pa.it (F.G.); marco.pensabene@policlinico.pa.it (M.P.); mariarita.dipace@policlinico.pa.it (M.R.D.P.)

**Keywords:** anorectal malformations, quality of life, long-term outcomes, bowel function, fecal incontinence, scores, transition of care, urinary function

## Abstract

**Background:** Anorectal malformations (ARMs) are rare congenital anomalies requiring surgical correction and long-term multidisciplinary care. Despite advances in surgical techniques like posterior sagittal anorectoplasty (PSARP), patients often experience ongoing issues with bowel and urinary function and psychosocial well-being. **Aim:** To evaluate the long-term outcomes of bowel function, urinary function, and quality of life in patients born with anorectal malformation and treated at our center. **Methods:** A cross-sectional study evaluated 55 ARM patients treated at the University Hospital of Palermo between 2002 and 2020. Data on clinical characteristics, surgical management, bowel and urinary function, and quality of life were collected using the following validated tools: Rintala Bowel Function Score, PedsQL Family Impact Module, Fecal Incontinence Quality of Life Score, and Lower Urinary Tract Symptoms Questionnaire. Statistical analysis was performed using Fisher’s exact test and ANOVA. **Results:** Excellent bowel function was observed in 44% of patients, particularly those with low-type ARMs. A bowel management program (BMP) was required in 62% of cases, though 44% of these patients, especially adolescents, showed poor adherence. BMP non-adherence significantly correlated with lower quality of life and worse bowel function (*p* < 0.01). Bladder dysfunction was noted in 24% of patients, mainly younger ones. **Conclusions:** Tailored BMPs and transition care are important for long-term success in ARM patients. Adherence to BMPs improves continence and quality of life, highlighting the need for continuous, multidisciplinary follow-up from childhood into adulthood.

## 1. Introduction

Anorectal malformations (ARMs) represent a diverse spectrum of congenital anomalies affecting the distal gastrointestinal tract. These malformations disrupt the continuity and functionality of the anorectal and perineal anatomy, requiring surgical correction and long-term multidisciplinary care. ARMs, with an incidence of approximately 1 in 4000 to 5000 live births, are frequently associated with other congenital abnormalities, particularly those involving the genitourinary system [1]. The condition’s complexity underscores the necessity for tailored clinical management strategies to optimize outcomes for affected individuals. Advances in surgical techniques, particularly posterior sagittal anorectoplasty (PSARP), introduced in the early 1980s, have significantly improved the anatomical and functional reconstruction of ARMs [2]. However, despite these advances, many patients continue to experience long-term complications, including fecal and urinary incontinence, constipation, and psychosocial challenges. Such challenges often extend into adolescence and adulthood, necessitating comprehensive follow-up and the implementation of bowel management programs (BMPs) to improve quality of life and social integration [3,4]. This study investigates the clinical outcomes, bowel function, urinary function, and quality of life of patients with ARMs treated at a tertiary care center. By evaluating long-term complications, adherence to BMPs, and psychosocial impacts, we aim to contribute to the growing body of literature focused on improving the care and support provided to this unique patient population. Our findings emphasize the role of bowel management and multidisciplinary care in meeting the changing needs of ARM patients from childhood through adulthood.

## 2. Materials and Methods

In November 2024, we conducted a retrospective study by a cross-sectional survey on patients born with anorectal malformations (ARM) who were treated at the Pediatric Surgery Unit of the “Paolo Giaccone” University Hospital of Palermo from 2002 to 2020. Demographic data were collected for each patient, and clinical records were used to gather information on the type of anorectal malformation, associated anomalies, type of surgical intervention, and postoperative complications. The type of malformation was classified using both the Krickenbeck classification and the International classification [5,6] (Table 1 and Table 2).

All patients who had completed toilet training by the time of the study were contacted by phone and were given questionnaires, sent via email, to evaluate the age at toilet training, the need for a bowel management program, adherence to therapy, bowel function, quality of life (personal or familiar), and urological symptoms in terms of bladder dysfunction. The parents were invited to complete the questionnaires on behalf of the young patients who were unable to provide responses independently. All patients with complete data were included. Bowel function was assessed using the Rintala Bowel Function Score, a questionnaire consisting of seven questions with values ranging from 0 to 3 for each question, except for the question on evacuation frequency, which has values from 1 to 2, resulting in a total score of 20. The Rintala Score is the only validated questionnaire in the literature, even for a population of healthy children. Patients with a score between 18 and 20 were considered to have “excellent” continence; those with a score between 13 and 17 were considered to have “good” continence; those with a score between 8 and 12 were considered to have “fair” continence; and those scoring below 8 were considered to have “poor” continence [7,8].

We decided to evaluate the impact of the condition on family quality of life in patients younger than nine years of age using the PedsQL 2.0 Family Impact Module. For patients aged nine years and older, we assessed quality of life related to fecal incontinence using the Fecal Incontinence Quality of Life (FIQL) score. Parents completed the PedsQL Family Impact Module by answering 35 questions. This validated tool is designed to assess the impact of pediatric chronic conditions on the family. It includes eight subdomains: physical, emotional, social, cognitive functioning, communication, concerns, daily activities, and family relationships. Each question was rated on a 5-point Likert scale (from ‘never a problem’ to ‘always a problem’) [9]. The final score and the individual domain scores were converted to a scale from 0 to 100, where a higher score indicates a lower impact of the child’s condition on family quality of life. Scores were grouped into four categories with thresholds indicating the degree of impact as follows:

- 75–100: Minimal Impact. The family experiences very low impact due to the child’s condition; overall family functioning is good.
- 50–74: Moderate Impact. The family faces some moderate effects related to the condition, with physical, emotional, or social limitations for parents and relatives.
- 25–49: Significant Impact. The family is significantly affected, with evident problems in physical, emotional, or social functioning impacting daily routines.
- 0–24: Severe Impact. The condition has a very serious impact on family life. Parents and relatives may face severe difficulties managing the situation.

In addition to the global assessment, a separate evaluation was conducted for each subdomain constituting the questionnaire.

The FIQLS comprises 29 questions used to evaluate four different aspects of quality of life related to fecal incontinence, including lifestyle, coping/behavioral adaptation, depression/self-perception, and embarrassment. Scores range from 1 to between 4 and 5 (depending on the domain), with lower scores indicative of poorer quality of life.

The final score and individual domain scores were converted to a scale from 0 to 100, where a higher score indicates a lower impact of fecal incontinence on quality of life [10]. Scores were divided into four categories with thresholds indicating the degree of impact as follows:

- 75–100: Minimal Impact. Quality of life is almost normal; fecal incontinence has little or no impact on emotions, behavior, and lifestyle.
- 50–74: Moderate Impact. The patient experiences some discomfort and limitation; condition management is good, but there are some effects on emotions and activities.
- 25–49: Significant Impact. Quality of life is significantly compromised, with major limitations in daily activities, social relationships, and self-perception.
- 0–24: Severe Impact. The condition severely affects quality of life, with substantial limitations and severe emotional and social distress.

Finally, bladder dysfunction was assessed in all patients using the Lower Urinary Tract Symptoms Questionnaire, a nine-question survey investigating the presence of bladder dysfunctions such as urinary incontinence, enuresis, and overactive bladder [11].

All patients enrolled for BMP underwent clinical evaluation to assess the presence of constipation or fecal incontinence. Constipated patients were managed with dietary modifications, laxatives, or enemas, based on individual clinical response. Patients with fecal incontinence were treated exclusively with enemas. Adherence to the prescribed treatment was assessed through self-reported information provided by the patients and/or their parents.

Despite the recognized importance of transition care for patients with congenital conditions reaching adulthood, our center lacks a standardized program where adult physicians manage these patients, leaving pediatric surgeons responsible beyond the pediatric age.

Statistical analysis was performed using Fisher’s exact test and ANOVA (for dichotomous variables), through a free software available online. Variables were considered statistically significant at the 5% level of significance (*p* < 0.05).

## 3. Results

From the review of clinical records, 77 patients born with anorectal malformations and treated at our center between 2002 and 2020 were identified. Of these, five died due to associated conditions, mainly severe congenital heart malformations, and another 17 could not be traced so they were excluded for incomplete data. Therefore, a total of 55 patients were included in the study and responded to questionnaires; of these, 29 (53%) were females. The average age of the sample was 12.7 years (4–22 years), with 11 patients (20%) aged younger than nine years and 44 patients (80%) aged nine years or older. Table 3 summarizes the types of ARM among the patients included in the study, classified according to the Krickenbeck and International classifications.

Twenty-five patients (45%) reported associated congenital conditions. Twenty-three patients (42%) underwent 3-stage surgery (colostomy–anorectoplasty–colostomy closure), nineteen patients (34%) underwent delayed primary PSARP, two patients (4%) underwent primary PSARP, and eleven patients (20%) underwent the cutback procedure. Only one patient still uses diapers due to a genetic syndrome associated with intellectual disability. Among the 54 patients who completed toilet training, the average age was 33 months (range 18–60 months).

Table 4 summarizes the average ages for toilet training according to the type of ARM (*p* = 0.00002, ANOVA test for the International classification).

Sixteen major and thirty minor complications were recorded. Among the major complications were the following:

• Six cases of anal stenosis: four underwent surgical stricturoplast; two were resolved with anal dilation using Hegar;
• Seven cases of neoanus mucosal prolapse were treated surgically;
• One anastomotic breakdown requiring redo PSARP;
• Two cases of abdominal obstruction: one due to fecaloma, treated conservatively; one due to adhesive bowel syndrome occurring nine years after the primary surgery, treated with exploratory laparotomy and visceral-parietal adhesiolysis;

Among the minor complications were the following:

• Twenty-three cases of perineal rash;
• Seven cases of perineal wound dehiscence treated conservatively.

From the collected medical history, 25 patients (45%) had issues related to constipation, while 9 patients (16%) were diagnosed with fecal incontinence. Among the 34 patients (62%) requiring a bowel management program, 15 (44%) reported non-adherence to prescribed therapies, and 11 (73%) of these non-adherent patients were aged between 9 and 18 years (*p* = 0.04, Fisher’s test).

Evaluation of the Rintala Bowel Function Score showed the following:

• Twenty-four patients (44%) had “excellent” continence, (8 M/16 F);
• Ten patients (18%) had “good” continence, (5 M/5 F);
• Seventeen patients (31%) had “fair” continence, (10 M/7 F);
• and four patients (7%) had “poor” continence, (3 M/1 F).

Table 5 summarizes the scores of all patients categorized by the type of ARM. Table 6 compares adherent and non-adherent patients to the BMPs with the Rintala Bowel Function Score (*p* = 0.02, Fisher’s test).

Analysis of the PedsQL 2.0 Family Impact Module revealed that

• Five families (46%) reported minimal impact on family quality of life;
• Four families (36%) reported moderate impact;
• and two families (18%) reported significant impact.

Regarding the FIQL score, 27 patients (61%) reported minimal impact on quality of life, nine (21%) reported moderate impact, and eight (18%) reported significant impact. No severe impacts were recorded on either family quality of life or quality of life related to fecal incontinence. Table 7 and Table 8 summarize the quality-of-life data, either family or personal, categorized by the type of malformation.

Table 9 shows the correlation between adherence to the BMP and its impact on quality of life (*p* = 0.0093, Fisher’s test).

A differentiated evaluation of the subdomains revealed significant differences, particularly in the behavior and embarrassment domains, when correlated with adherence to BMPs. Non-adherent patients had an average score of 39/100 and 42/100 in the behavior and embarrassment domains, respectively, while adherent patients had average scores of 72/100 and 82/100 (*p* < 0.01 in both cases, ANOVA test).

Finally, 13 patients (24%) (4 M/9 F) exhibited symptoms of bladder dysfunction, nine (69%) of whom were aged 13 years or younger. Nine patients presented overactive bladder symptoms while four patients presented nocturnal enuresis.

## 4. Discussion

The advent of posterior sagittal anorectoplasty, described by Peña in 1982, modernized the surgical approach to this type of malformation, characterized by a spectrum of different clinical conditions. It allowed direct visualization of recto-urogenital communications, ensuring better anatomical and functional reconstruction [2]. Improvements in perioperative and postoperative care, as well as in managing associated anomalies, have drastically reduced the mortality rate of patients with ARMs. Today, an increasing number of patients face the long-term consequences of a congenital condition [12]. In recent years, scientific research has increasingly focused on the long-term problems faced by these patients, especially concerning bowel function and quality of life [3,4]. Brisighelli et al. reported that 44% of patients with low ARMs, classified according to the International classification, had excellent bowel function as assessed by the Rintala Bowel Function Score, while only 8% of patients with intermediate ARMs achieved similar outcomes [13]. Similar data were reported by Rintala in his population [7,8]. In our series, we recorded slightly better outcomes in patients with low ARMs, with 69% demonstrating excellent bowel function. Additionally, the percentage of patients with intermediate ARMs and excellent bowel function, also 8%, was confirmed in our population.

Many patients born with ARMs experience fecal incontinence, soiling, or severe constipation after surgery and completing toilet training. According to the literature, up to 79% of ARM patients experience constipation, and approximately 48% report episodes of soiling, requiring an appropriate bowel management program to achieve socially acceptable continence and maintain a good quality of life [14,15,16]. Similarly, in our cohort, nearly 50% of the analyzed sample suffered from constipation.

The complexity of ARMs significantly impacts fecal and urinary continence, as well as psychosocial aspects, which vary across different growth stages. Psychosocial difficulties can arise as early as childhood and tend to intensify with age, particularly if not adequately addressed. These challenges are often exacerbated during adolescence and the transition to adulthood, which are critical periods for emotional and cognitive development [17]. Numerous studies underscore the importance of incorporating quality-of-life assessments into the monitoring of these patients, serving as essential tools for a comprehensive analysis of their overall conditions [18,19,20,21]. A study by Trinidad et al. highlighted how fecal incontinence significantly impacts all analyzed quality-of-life domains, with the most significant impact observed in the areas of behavior/adaptation and embarrassment [22]. Our work revealed similar findings, especially among patients who did not adhere to bowel management programs.

ARMs are often associated with urological conditions affecting both the upper and lower urinary tracts. The close association with urological malformations necessitates neonatal screening for renal pathologies, though less attention is given to bladder dysfunctions that may manifest in early childhood or adulthood. According to the literature, urinary incontinence incidence across ARM types ranges from 1.7% to 41%, depending on the cohort [21,22,23]. Similarly, bladder dysfunction symptoms were observed in 24% of patients in our cohort.

This variability may be explained by the fact that urinary incontinence is less stigmatized in society, with readily available incontinence products reducing its impact on patients’ lives to the extent that it is almost considered a normal condition [11].

Patients with ARMs can develop complications such as fecal and urinary incontinence, constipation, ejaculatory or erectile dysfunction, and psychological problems, all of which affect their quality of life. Many of these issues become apparent later in life, especially during adolescence [21]. Therefore, extending the follow-up of these patients and establishing multidisciplinary teams to address the challenges that arise over time should be desirable. Our data show that dedicated BMPs for each patient improve outcomes in terms of quality of life and bowel function. Adolescents represent a high-risk population with a greater likelihood of loss to follow-up and lower adherence to BMPs, leading to deteriorated bowel function and poorer quality of life. Despite advances in pediatric surgery, many ARM patients experience persistent symptoms into adulthood, making the transition care into adulthood more important [20,24]. Transition care should begin around ages 12–13 and be completed between 18 and 21. The transition phase is not merely a transfer of care but a preparation process that includes educating patients and their families on long-term disease management [21]. A well-organized and continuous transition improves symptom control, therapy adherence, and patient satisfaction with care received [20,21,24].

The limitations of the study include a relatively small sample size, particularly for certain subgroups of anorectal malformations, and the absence of multivariate data analysis. Moreover, the adequacy to BMP is closely related to the compliance of both the patient and their families. We assumed that all information reported is reliable, without any intentional misreporting.

In conclusion, BMPs are fundamental in the long-term management of ARM patients to improve outcomes. While surgical interventions provide anatomical correction, BMPs are essential for ensuring functional success and improving quality of life. We should encourage the establishment of transition care programs to optimize specific needs that may arise during the shift from pediatric to adult care, focusing on medical management and psychosocial well-being.

## Figures and Tables

**Table 1:** Krickenbeck classification.

Major Clinical Groups	Rare Regional Variants
Perineal	Pouch colon
Rectourethral fistula -Prostatic -Bulbar	Rectal atresia/Stenosis
Rectovesical fistula	Rectovaginal fistula
Vestibular fistula	H type fistula
Cloaca	Others
No fistula	
Anal stenosis	

**Table 2:** International classification.

	Female	Male
High	Anorectal agenesis A: Rectal atresia B: With fistula Rectocloacal fistula Rectovaginal/high Rectal atresia	Anorectal agenesis A: Rectal atresia B: With fistula Rectovesical fistula Rectourethral fistula Rectal atresia
Intermediate	Anal agenesis A: Without fistula B: With fistula Rectovaginal low Rectovestibular fistula Anorectal stenosis	Anorectal agenesis A: Without fistula B: With fistula Rectobulbar fistula Anorectal stenosis
Low	At normal anal site Covered anus—complete Covered anal stenosis Rectal atresia Anocutaneous fistula Anterior perineal anus At vulvar site Vulvar anus Anovulvar fistula Anovestibular fistula	At normal anal site Covered anus—complete Covered anal stenosis Rectal atresia Anocutaneous fistula Anterior perineal anus
Miscellaneous	Anal membrane stenosis Imperforated anal membrane Perineal groove Perineal canal	Anal membrane stenosis Imperforated anal membrane Perineal groove Perineal canal

**Table 3:** Patients included in the study are divided by type of ARM according to the International and Krickenbeck classification.

Type of ARM	N° Patients (n = 55)	%
Krickenbeck classification		
Perineal	25	45
Vestibular	14	25
Bulbar	5	9
Prostatic	5	9
Bladder-neck	3	6
Imperforate anus without fistula	3	6
International classification		
Low	33	60
Intermediate	12	22
High	10	18

**Table 4:** Median age at potty training according to the type of ARM according to Krickenbeck and International classifications (for the International Classification: *p* = 0.00002 with ANOVA) (n = 55).

	Potty Training Age in Months (Range)
Krickenbeck classification	
Perineal	30 (18–60)
Vestibular	30.7 (18–48)
Bulbar	37.6 (26–48)
Prostatic	40.4 (24–48)
Bladder-neck	44 (36–60)
Imperforate anus without fistula	42 (36–48)
International classification	
Low	29.4 (18–60)
Intermediate	36.6 (24–48)
High	42.4 (26–60)

**Table 5:** Results of the Rintala Bowel Function Score divided by type of ARM according to the Krickenbeck and International classification (n = 55).

Type of ARM	Excellent (18–20)	Good (13–17)	Fair (8–12)	Poor (<8)
Krickenbeck classification				
Perineal	16	4	4	1
Vestibular	7	3	4	
Bulbar		1	2	2
Prostatic		1	4	
Bladder-neck		1	2	
Imperforate anus without fistula	1		1	1
International classification				
Low	23	5	4	1
Intermediate	1	3	6	2
High		2	7	1

**Table 6:** Rintala Bowel Function Score results of patients requiring bowel management (*p* = 0.02, Fisher’s test) (n = 34 all patients on BMP).

Adherence to BMPs	Excellent	Good	Fair	Poor
Not adherence	0	1 (7%)	11 (73%)	3 (20%)
Adherence	6 (32%)	6 (32%)	6 (32%)	1 (4%)

**Table 7:** Results of the PedsQL Family Impact Module divided by type of ARM according to the Krickenbeck and International classification (n = 11 patients younger than 9 years).

Type of ARM	Minimal	Moderate	Significant	Severe
Krickenbeck classification				
Perineal	3	1		
Vestibular	2	1	1	
Bulbar				
Prostatic		1		
Bladder-neck		1		
Imperforate anus without fistula			1	
International classification				
Low	4	1		
Intermediate	1	1	1	
High		2	1	

**Table 8:** Results of the FIQL score divided by type of ARM according to the Krickenbeck and International classification (n = 44 9 years or older).

Type of ARM	Minimal	Moderate	Significant	Severe
Krickenbeck classification				
Perineal	17	3	1	
Vestibular	8		2	
Bulbar		1	4	
Prostatic	1	2	1	
Bladder-neck		2		
Imperforate anus without fistula	1	1		
International classification				
Low	24	3	1	
Intermediate	2	1	6	
High	1	5	1	

**Table 9:** FIQL score results of patients requiring bowel management (*p* = 0.0093, Fisher’s test) (n = 27 9 years or older).

Adherence to BMPs	Minimal	Moderate	Significant	Severe
Adherence	9 (64%)	4 (29%)	1 (7%)	
Not adherence	1 (8%)	5 (38%)	7 (54%)	

## Data Availability

The data presented in this study are available from the corresponding author upon request due to privacy.
